# Chatbots’ Empathetic Conversations and Responses: A Qualitative Study of Help‑Seeking Queries on Depressive Moods Across 8 Commercial Conversational Agents

**DOI:** 10.2196/71538

**Published:** 2025-11-24

**Authors:** Hyojin Chin, Gumhee Baek, Chiyoung Cha, Meeyoung Cha

**Affiliations:** 1 Department of Computer Science and Engineering, Gyeongsang National University, Jinju, Republic of Korea; 2 College of Nursing, System Health Science and Engineering Program, Ewha Womans University, Seoul, Republic of Korea; 3 College of Nursing, Ewha Research Institute of Nursing Science, System Health and Engineering Major in Graduate School, Ewha Womans University, Seoul, Republic of Korea; 4 Max Planck Institute for Security and Privacy, Universitätsstraße 140, Bochum, 44799, Germany, 49 55-772-1387, 49 55-772-3329; 5 School of Computing, Korea Advanced Institute of Science and Technology, Daejeon, Republic of Korea

**Keywords:** chatbot, conversational agent, depressive moods, depression, help-seeking, therapeutic communication, social support, qualitative study

## Abstract

**Background:**

While recent studies showed the potential of conversational agents (CAs) to help alleviate depressive moods, the dynamics of user-chatbot interactions in mental health support remain underexplored.

**Objective:**

We examine real-world conversations between users and chatbots on depression-related topics to identify patterns in how users seek help and how chatbots provide therapeutic support. We analyzed the responses of 8 commercial chatbots to user queries about depressive moods, examining whether they incorporated therapeutic communication techniques, such as empathy.

**Methods:**

Our method has 2 parts. First, we analyzed 13,700 utterances (6850 user queries and 6850 responses) about depressive moods from the commercial chatbot SimSimi, covering 5 English-speaking countries between 2016 and 2021. Using a human-annotated coding approach, we classified user queries into 5 groups based on Rickwood’s help-seeking model and classified chatbot responses into 8 therapeutic communication styles. Empathy was assessed as one of these styles, with responses coded as empathetic when they demonstrated emotional understanding, validation, and reflection. Next, we evaluated the responses of 3 voice assistants (Amazon’s Alexa, Google Assistant, and Apple’s Siri) and 5 chatbots (ChatGPT, Replika, Woebot, Wysa, and SimSimi) to user queries about depressive moods.

**Results:**

In study 1, we examined how SimSimi, a social chatbot trained to encourage users to share their emotions and build rapport, responded to user queries. The majority (3067/4073, 75.3%) indicated depressed feelings, and a smaller portion (168/4073, 4.1%) sought strategies to cope with depression. The chatbot’s responses were largely therapeutic ( 2417/3108, 77.7%), demonstrating empathy (902/3108, 29%), active listening (836/3108, 26.9%), and open-ended questions (679/3108, 21.8%). In study 2, we qualitatively compared response patterns across commercial CAs, revealing that Replika expressed empathy in more than 75% (28/36) of its responses, similar to SimSimi. In contrast, Alexa (15/17, 88.2%), Google Assistant (18/30, 60%), Siri (20/36, 55.6%), and ChatGPT (40/42, 95.2%) typically responded to depression-related queries with search results rather than offering specific solutions for depressive feelings. Mental health chatbots such as Woebot responded to users with clarification questions (97.3%). We also report instances where CAs failed to meet users’ help-seeking needs, instead giving irrelevant responses and ignoring emotional requests.

**Conclusions:**

Our findings reveal a mixed landscape in the emotional support provided by CAs. While some social chatbots delivered empathetic responses that fostered deeper user engagement, most commercial chatbots offered merely informative replies to users’ help-seeking inputs. Recognizing that users seek support from chatbots, we recommend equipping next-generation CAs with capabilities grounded in therapeutic communication, such as empathetic responses.

## Introduction

### Background

Depression is a severe illness that affects around 280 million people globally [1]. The COVID-19 pandemic worsened the situation, with an estimated 27.6% increase in cases of major depressive disorder [2]. Individuals with depression symptoms may avoid seeking medical help due to cost, time, possible side effects of medication, and social stigma [3]. The resulting unmet need for depression support has increased the demand for alternative services. For example, the number of downloads of online mental health apps increased by 17.6% after the pandemic [4,5], indicating a public need for easy-to-access mental support.

Chatbots are gaining popularity in the mental health domain, with studies showing their positive effects on reducing depression, stress, and anxiety [6,7]. Research suggests that many individuals are more willing to engage with artificial intelligence (AI) agents, such as chatbots, because of the anonymity they offer and the perception of a “safe space” for discussing sensitive issues without fear of judgment [8]. This report highlights that AI agents can serve as an alternative to human counselors for those struggling with depression and anxiety, especially in cultures where mental health stigma is prevalent [9,10]. In addition, chatbots offer users 24/7 social support and can facilitate mental health management [11].

In response, the industry has swiftly developed commercial chatbots such as Woebot, Wysa, and Vivibot, which provide cognitive behavioral therapy (CBT), behavioral reinforcement, and mindfulness [11]. A study examining the AI-based mobile conversation app, Wysa, indicated that therapeutic alliance scores improved with increasing conversation time [12]. Furthermore, research on Tess, a chat-based app that offers emotional support and psychological education, demonstrated a significant reduction in user anxiety upon engagement [13]. An analysis of 152,783 conversations related to depressive moods and sadness from 4 years and 8 months of conversation data between commercial chatbots and users revealed that users were more open to expressing emotional vulnerability related to depressive or sad moods to chatbots than on social media [14].

Despite the exciting potential of AI chatbots as a source of support in mental health domains, most research has focused on technical functionalities [15] and the outcome of user interactions (eg, depression reduction) [12,16]. Some studies focused on mental health issues have used discourse analysis to examine data from platforms such as Twitter and Reddit [17,18]. Other research has analyzed user discourse, revealing distinct patterns of depressive mood expression among social chatbot users compared with social media users, with a tendency to disclose depressed and sad emotional states to chatbots [14]. These studies suggest that valuable insights can be gained from examining user queries. However, what is currently lacking is a discourse-based analysis of conversational AI systems to explore how they might respond therapeutically to diverse user needs related to depressive moods.

In this study, we address the gap by examining how users with depressive moods seek help from chatbots and how various commercial chatbots respond therapeutically. We pose the following research questions: (RQ1) How do users initiate help-seeking conversations related to depressive moods with online chatbots? (RQ2) Do commercial conversational agents (CAs) respond therapeutically to help-seeking user input about depressive moods, and if so, how? These research questions are important because they provide complementary perspectives on help-seeking interactions. While RQ1 focuses on understanding how users with depressive moods initiate emotional disclosures, RQ2 explores how commercial CAs respond to such expressions. This understanding is critical for designing chatbots that can effectively recognize and respond to users’ emotional states. In addition, comparing the diverse responses across commercial products is important for assessing their current capabilities and limitations.

We partnered with SimSimi, one of the largest chatbot services, to categorize help-seeking behaviors related to depression. We then conducted our experiments with 8 commercial CAs: 3 voice assistants (Amazon’s Alexa, Google Assistant, and Apple’s Siri) and 5 chatbots (ChatGPT, Replika, Woebot, Wysa, and SimSimi). Users today frequently encounter a variety of CAs in everyday life, accessed through different modalities. For example, they may interact with Alexa via a stationary smart speaker at home, use Siri on a smartphone, or engage with personalized chatbots such as SimSimi or Replika through mobile apps. In addition, users may choose to converse with advanced AI systems such as ChatGPT or similar large language model (LLM)–based chatbots offering high-performance dialogue capabilities. However, in this study, we did not delve into the differences in interaction modalities (eg, text vs voice) or the technical performance variations across different AI models. Instead, we selected candidate CAs based on overall usability and accessibility from the user’s perspective, aiming to reflect general user experiences with a diverse yet representative range of CAs.

Our findings reveal that while some of these services assist users by suggesting solutions, asking clarifying questions, and offering empathetic responses, others struggle to meet users’ help-seeking needs. We observed instances where services failed to adequately address user requests, generating irrelevant responses that neglected users’ emotional needs. Given that most current CAs primarily provide information, a crucial goal is to develop empathetic CAs capable of using therapeutic communication methods to effectively support users’ social needs. Our findings offer critical evidence on the type of assistance users expect from chatbots and provide valuable insights for future chatbot designs.

### Preliminaries

#### Chatbots and Mental Health

The widespread adoption of social media has enabled people to easily express their thoughts and emotions [19]. Studies have shown that expressing emotions on social media can reduce depression, anxiety, and loneliness while increasing overall life satisfaction by helping individuals cope with negative emotions [20]. Social media usage patterns are also known to signal mental health status. For example, Facebook users with depression or anxiety symptoms tend to spend much time on the platform and engage in passive behaviors, such as repeatedly browsing other users’ profiles [21]. Meanwhile, WhatsApp and Instagram users ruminate on negative thoughts without interaction [22]. As previous studies have demonstrated, online user-generated content can be a valuable resource for understanding the psychological states of users [23,24].

Chatbots offer users 24/7 social support, and they have been shown to facilitate mental health management [11]. Some chatbots are designed for therapeutic functions and offer psychological support based on acceptance and commitment therapy and CBT [25]. Chatbots that are specifically designed to alleviate depression provide a therapeutic virtual space in which they offer one-on-one psychological support while incorporating both CBT and acceptance commitment therapy technologies; such chatbots are thus more effective than general chatbots in mental health management [14,26]. A study on the users of Wysa, the AI-based emotional intelligence mobile conversation agent app, found that although the Working Alliance Inventory-Short Revised scores were not comparable with the face-to-face treatment scores, the therapeutic alliance scores improved with increasing conversation time between the chatbot and users [14]. However, the overall effectiveness remained subpar relative to face-to-face treatment scores. Another study on a chat-based app that offers emotional support and psychological education, Tess, showed a significant anxiety reduction [25]. However, the study noted that app usage did not substantially affect decreasing depressive symptoms. Another study found that although users may make errors when using modules with diverse designs, they generally have a natural affinity for chatbots [6].

Prior studies have demonstrated that psychological support chatbots can effectively alleviate anxiety and depression; however, their effectiveness is limited by engagement and the tendency to focus primarily on conversations related to depression [10,11,14,27]. Furthermore, the analysis of agent responses has often been overlooked in existing research. Some studies have examined the interaction data between users and chatbots, but many have concentrated on machine learning–based autodetection [28] or have been limited to COVID-19 data [29,30]. Analyzing chatbot-user conversations to understand the effects of chatbots as a supportive form of psychotherapy can offer valuable insights for the advancement of chatbot research in the mental health domain.

#### Categories of Help-Seeking Disclosure Patterns

Help-seeking involves searching for external support to deal with problems [31]. Among the various help-seeking models, Rickwood’s model—focused on the stages of mental health help seeking—was selected for its applicability to online interactions, as it outlines 4 key stages: recognizing symptoms, acknowledging the need for help, communicating symptoms, and finding a source of supporting information [26]. Based on this model, we categorized users’ discourse into six themes: (1) isolation and loneliness [27], (2) difficulties in relationships and communication [28,29], (3) depressed feelings [6,26], (4) strategies for dealing with depression [11,26], (5) disclosure of depression diagnosis [30], and (6) irrelevant utterances. Table 1 provides definitions for these 6 categories, each representing a unique pattern of user intent and emotional state during chatbot interactions.

**Table 1.** Definitions of 6 categories of help-seeking discourse patterns related to depressive mood.

Category	Definition
Isolation and loneliness	Wanting to be with others because you are lonely or help-seeking so that you are not lonely.
Difficulties in relationships and communication	Help-seeking because of depressive mood caused by others (family, friends, etc).
Depressed feelings	Help-seeking by revealing depressed feelings (eg, “I’m sad” or “I’m not in a good mood”).
Strategies for dealing with depression	Help-seeking to find ways to reduce depressive mood (therapy, medications, etc).
Disclosure or revelation of a depression diagnosis	Help-seeking after disclosing being diagnosed with depression.
Irrelevant utterances	Difficulty grasping content or others (user is not depressed).

#### Therapeutic Communication of Chatbots

Therapeutic communication is a mental health approach that helps individuals express their emotions and address personal needs through supportive interactions [32-34]. In this study, we conceptualize emotional support as the overarching function that chatbots may provide to users experiencing depressive moods. Within this framework, therapeutic communication refers to the structured method through which such support is delivered. Building on Goldin and Russell’s foundational elements—such as empathy, active listening, and encouragement [35]—this concept has been widely applied in health care settings. Sharma and Gupta [36] proposed 14 techniques for therapeutic communication. We used these 14 definitions to identify categories of therapeutic communication. We excluded 5 categories unsuitable for a chat context (ie, establishing a genuine therapeutic relationship, respecting privacy, using formal titles, starting with social pleasantries, and summarizing) and used the remaining 9 categories as evaluation measures (Table 2). Each reflects a unique way in which chatbots may foster therapeutic engagement during user interactions.

**Table 2.** Definitions of 9 therapeutic communication categories.

Category	Definition
Open-ended questioning	Approach involving questions that encourage users to provide detailed and descriptive answers.
Active listening techniques	Paying full attention to user requests, with a focus on active listening and careful attention to user needs.
Nonverbal and verbal cues	Verbal and nonverbal cues, including statements such as “Uh-huh” or “I see,” show that the chatbot understands and acknowledges the user’s message.
Empathetic responses	Demonstration of empathy and validation and reflection on a user’s feelings.
Silence	Demonstration of attentiveness by minimizing interruptions to the user.
Clarification question	Providing guiding statements to help the user clarify statements and to obtain additional information.
Provide solution	Providing practical solutions for users’ help-seeking related to depression.
Nonverbal communication	In human-to-human communication, emphasis is placed on using body language and facial expressions to convey messages; by contrast, chatbots use emoticons.
Irrelevant answers	Irrelevant responses, nontherapeutic communication, provision of unverified information, etc.

Therefore, the aim of this study is to investigate how users with depressive moods seek help through online chatbots and to evaluate the extent to which commercial CAs respond empathetically and therapeutically to such help-seeking input.

## Methods

### Research Overview

This study is reported in accordance with the Standards for Reporting Qualitative Research [37]. We conducted 2 qualitative studies, analyzing data from 13,700 anonymized real-world chatbot-human conversations (6850 user queries and 6850 responses) on depressive mood from users in 5 countries (Canada, Malaysia, Philippines, United Kingdom, and United States) using SimSimi, a popular chatbot with more than 400 million users in 111 languages. These data offer a unique perspective on user-AI chatbot interactions from both the user and the machine side. Study 1 categorizes user messages based on Rickwood’s help-seeking model (see 6 categories in Table 1) and classifies chatbot responses into 9 therapeutic communication styles adapted for chatbot communication using techniques proposed by Sharma and Gupta [36] (Table 2).

**Figure 1.**
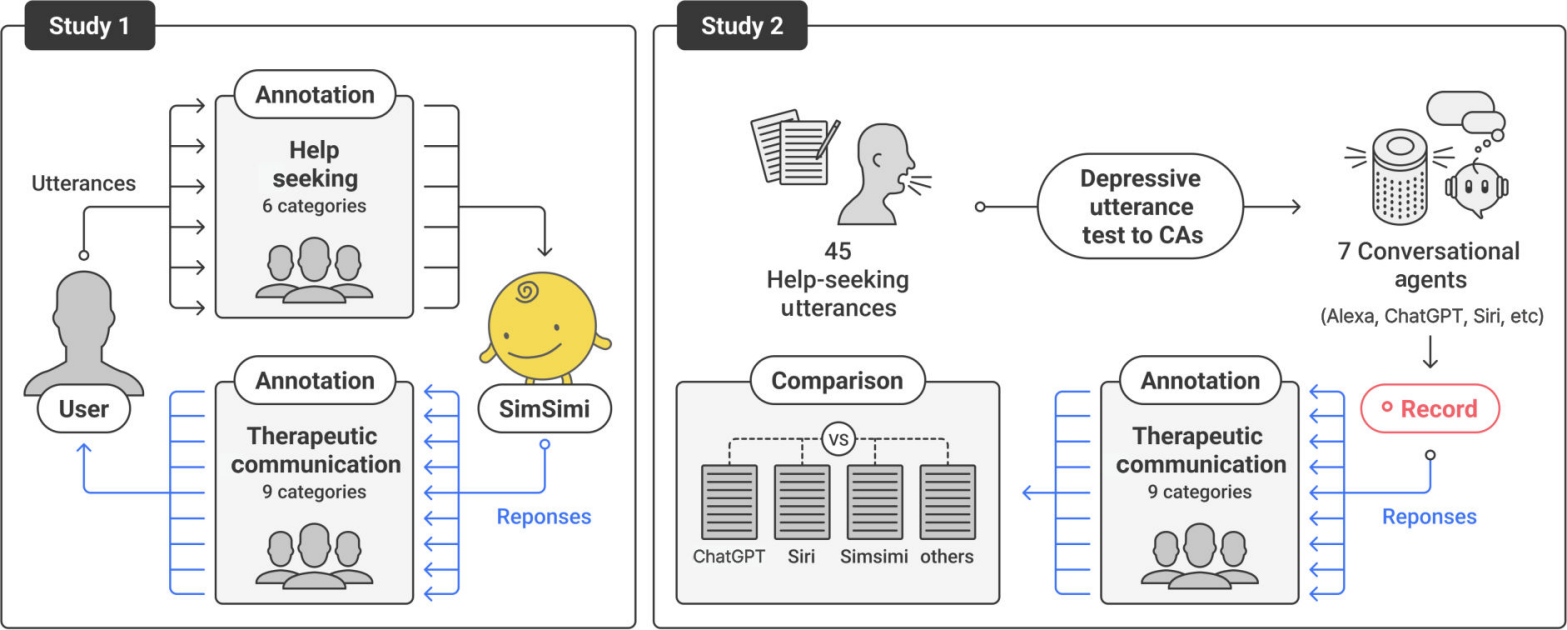
Study design. CAs: conversational agents.

We also broadened the medium to a broader set of chatbot platforms and compared how commercial CAs handle depression-related concerns. Our study 2 explores the support offered by 5 chatbots (ChatGPT, Replika, Woebot, Wysa, and SimSimi) and 3 voice assistants (Amazon’s Alexa, Google Assistant, and Apple’s Siri) by sending them messages based on the categories of help-seeking identified in our initial study. The responses of each of the 8 CAs were collected and analyzed to compare the types of support they provided (Figure 1).

### Study 1: Users’ Depressive Mood–Related Help-Seeking Using Chatbots and Chatbots’ Therapeutic Responses

#### AI Chatbot SimSimi

Study 1 aimed to determine how individuals communicate their depressive moods to SimSimi, a widely used chatbot that facilitates social interaction and entertainment, and to examine its responses. SimSimi was launched in 2002 and has amassed more than 400 million users globally, with up to 200 million chat utterances recorded daily in 111 languages.

**Figure 2.**
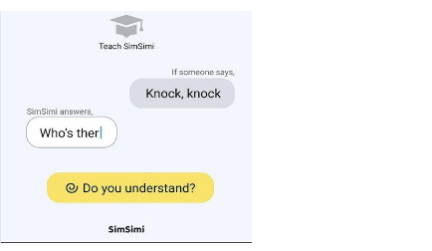
SimSimi’s “teach” function is an example of how the chatbot seeks to improve its responses. In cases where it cannot answer a question, it asks users for input, allowing them to teach SimSimi the answer they think is correct or prefer to hear.

SimSimi differentiates itself from other chatbots by offering a “teach SimSimi” function that instructs users on how to answer specific questions they pose [38] (Figure 2). When SimSimi encounters an unanswered question, it explicitly asks the user for input. Users can teach SimSimi the answers they want to hear or believe to be correct, and these responses are added to the main database through crowdsourcing. Currently, SimSimi’s database contains more than 115 million conversation pairs, which are searched by the service to generate the most appropriate responses to user inputs.

Examining this particular social chatbot offers several benefits. First, SimSimi is a mobile app, making it easily accessible to users in private settings. Its high accessibility ensures anonymity and encourages active user engagement on sensitive topics. Second, unlike task-oriented CAs, such as Amazon’s Alexa and Apple’s Siri, SimSimi is an open-domain chat service that resembles a general conversation. This feature is essential for understanding the natural need for social support in everyday conversations. Finally, SimSimi is among the most frequently downloaded chatbot services globally.

The data used in this study were obtained from SimSimi Inc. These data included user queries, the chatbot’s responses, time stamps, the unique user IDs generated by the app, and country information. SimSimi obtains geolocation information via the time zone settings of users’ devices; this information is then linked to a specific country in the database. SimSimi safeguards user privacy by not collecting or storing personally identifiable information (PII); thus, the data used in this study did not include PII, such as username, age, gender, address, social security number, or phone number. The company uses internal text-filtering methods to protect PII even when users disclose such information during conversations (eg, by replacing consecutive numbers with strings of asterisks).

#### Dataset

Anonymized data were collected from SimSimi, focusing on users who expressed depressive mood–related words in English over 2078 days between May 1, 2016, and December 31, 2021. This period includes the COVID-19 pandemic, during which individuals faced various challenges, including a significant reduction in social interaction due to lockdowns and quarantine measures. Depression and loneliness were widely reported to have increased during this time, making it a meaningful period for examining help-seeking behavior through chatbots. We excluded users who reported being clinically depressed or having other psychiatric disorders. We limited our data to users declaring their residency in 5 English-speaking countries: Canada, Malaysia, Philippines, United Kingdom, and United States. We used 2 steps to extract our data. First, we extracted 564,191 sentences that contained keywords associated with depressive moods, such as “depress,” “distress,” “dejected,” “gloomy,” “cheerless,” “sad,” “feeling low,” “hate myself,” and “ashamed of myself” [39]. Keyword mapping can lead to false positives (eg, “economic depression”). Therefore, in the second step, referring to the *Diagnostic and Statistical Manual of Mental Disorders* (*Fifth Edition*) (*DSM-V*) [40] and the World Health Organization’s Mental Health information web page [41], 2 health professionals compiled the keywords listed in Textbox 1 and selected 6850 conversation datasets that consisted of 1 user utterance and 1 chatbot answer each (Table 3).

Textbox 1. **Keywords for extracting utterances of depressive mood–related help-seeking.**

advice, adviser, aid, assist, behavior, care, CBT, cognitive behavior therapy, check, cognitive, consult, control, counsel, counselor, cure, diagno, disease, disorder, doctor, drug, DSM, diagnostic and statistical manual of mental disorder, ECT, electroconvulsive therapy, electro, emotion, empathy, evaluation, exam, examin, help, hospital, ill, illness, lab, magnetic, medication, medicine, mental, mood, need, neurosis, nurse, pill, practitioner, program, psych, rehabilitation, remedy, resolve, shrink, sick, sign, solution, stimulation, support, symptom, tablet, therap, therapist, TMS, transcranial magnetic stimulation, transcranial, treat.

**Table 3.** Number of final extracted data by year.

Year	Values, n
2016	2647
2017	1702
2018	663
2019	782
2020	623
2021	433
Total	6850

#### Data Annotation

To identify the different types of depression-related help-seeking that users directed to SimSimi and understand how SimSimi provided therapeutic communication, we categorized 13,700 datasets, which included 6850 user queries and 6850 responses from SimSimi. Chats involving depression-related help-seeking were classified into six categories based on Rickwood’s help-seeking model: (1) isolation and loneliness, (2) difficulties in relationships and communication, (3) depression, (4) strategies for dealing with depression, (5) disclosure or revelation of a depression diagnosis, and (6) irrelevant utterances.

We classified therapeutic communication responses from the chatbot using nine categories from Sharma and Gupta’s therapeutic communication model [36], which were modified to fit the context of chatbots: (1) open-ended questioning, (2) active listening techniques, (3) nonverbal and verbal cues, (4) empathetic responses, (5) silence, (6) clarification questions, (7) providing solutions, (8) nonverbal communication; and (9) irrelevant answers.

Data labeling was performed by 1 researcher and 5 students with a medical background. To achieve a precise annotation of help-seeking user chats and the chatbot’s therapeutic communication response type, we provided detailed guidelines and conducted an in-depth training session for all annotators. The training included two sessions: (1) an initial 2-hour orientation on coding guidelines and category definitions, and (2) a calibration session where annotators independently coded 100 sample utterance-response pairs. This process was designed to identify and discuss ambiguities in advance and ensure a shared understanding of labeling criteria. Following training, annotators independently labeled the full dataset. Three annotators classified the 6850 user queries into help-seeking categories while another set of 3 annotators classified the 6850 responses from SimSimi into therapeutic communication categories. This phase was performed over a period of 4 weeks. During the annotation process, annotators were encouraged to raise ambiguous or difficult cases, which were then discussed in weekly meetings with the researcher. In cases of disagreement, annotators shared their differing interpretations and reached a consensus through group discussion. Only the data that were agreed upon by all annotators were included in the final results. Given the potentially distressing nature of some user utterances, annotators were informed in advance about the possibility of experiencing emotional discomfort during the annotation process. They were encouraged to take breaks as needed and to raise any concerns during weekly check-in meetings. In addition, a list of mental health support resources was provided, including the National Center for Mental Health, which offers emergency consultation services and mental health support. Fleiss’ κ [42] was used to measure the reliability of annotations. The total number of data points considered, excluding the eliminated data, was 6108 for the users and 6150 for SimSimi; the Fleiss’ κ values for the chat classification were 0.87 and 0.89, respectively.

### Study 2: CAs’ Responses to Users’ Depressive Mood–Related Help-Seeking

#### Selecting Candidate CAs

In study 2, we examined how commonly used AI-powered CAs respond to users seeking help for depression and assessed their response patterns. We ran a depressive mood utterance test and compared the therapeutic responses of 8 CAs, including SimSimi, in relation to the 5 categories of help-seeking related to depression identified in study 1. Procedures for testing depressive utterances with CAs are described in the following subsections.

We selected 7 CAs, comprising 3 voice assistants and 4 chatbots. We aimed to include at least 2 CAs from each category—task-oriented agents, social chatbots, and mental health chatbots—to examine the variance in their response methods. Our selection was based on general usability and accessibility from a user perspective, disregarding differences in modality between voice and text.

We included Amazon’s Alexa, Google Assistant, and Apple’s Siri, which are widely used voice assistants and task-oriented CAs according to the Voice Consumer Index 2022 [43]. These assistants are designed to interact with users through voice commands and speech.

In addition, we selected 4 widely used chatbots, ChatGPT, Replika, Woebot, and Wysa, all of which interact with users through text-based chat interfaces such as messaging apps or website chat windows. ChatGPT, a task-oriented CA, was selected as a representative example among LLM-based chatbots, as it was the most widely used and publicly accessible model at the time of study design [44] . In contrast, Replika is a social chatbot with traits similar to SimSimi [45,46]. Wysa and Woebot differ slightly from these CAs: they were specifically developed for mental health and offer a range of techniques, including CBT, mindfulness, and dialectical behavior therapy [10-12]. They may also provide distinct solutions and responses, unlike SimSimi and Replika, which are used for casual conversations. All these CAs can be easily accessed on mobile phones or personal computers without additional equipment. Therefore, we chose these 7 CAs and compared their differences and tendencies with the responses of SimSimi, which we examined in study 1.

#### Depressive Utterance Test for CAs

We conducted a depressive utterance test on the 7 CAs to investigate how commercially available chatbots and voice assistants respond to user queries about depressive mood. We followed a testing method similar to a previous study that examined how CAs respond to users who use abusive language [47]. First, we selected 3 sentences from the “User utterance examples” in Table 4 that best represented each of the 5 categories of help-seeking identified in study 1. We then used these 15 sentences as inputs for each of the 7 CAs and tested each sentence 3 times. Hence, 45 queries were tested in total. We tested the voice assistants (Amazon’s Alexa, Google Assistant, and Apple’s Siri) by directly stating selected depression-related sentences to them and then recording their responses. For the chatbots (ChatGPT, Replika, Woebot, and Wysa), we sent the help-seeking sentences as messages and recorded their responses. While the set of input sentences used in this stage was limited in size, it was constructed to capture a balanced range of representative help-seeking categories. Although modest in scale, the design was deliberately structured to allow for a systematic and comparative analysis across 7 different CAs. This focused yet efficient experimental framework facilitated the identification of distinct response patterns and stylistic tendencies in how various CAs handle expressions of depressive mood. Testing was conducted by 2 researchers and 2 medical students. We then collected and transcribed the responses of each CA.

**Table 4.** Frequency of users’ depression-related help-seeking discourse by category and examples of user utterances.

User categories	Distribution, n (%)	Example user utterances
Depressed feelings	3067 (75.3)	I feel depressed. Can you help me :( I’m sad. I need your hug. Can you help me feel less sad?
Difficulties in relationships and communication	470 (11.5)	No one cares about me...my family? So sad they don’t like me. I’m sad. My friends don’t care about me. I’m sad. Dad is always angry, and mom doesn’t help.
Isolation and loneliness	236 (5.8)	I am lonely. I need you. I’m lonely so I need someone. Help me. I have no one to talk to.
Strategies for dealing with depression	168 (4.1)	What is the best cure for depression? How do you cure depression? How to control depression?
Disclosure or revelation of a depression diagnosis	132 (3.2)	I am currently experiencing mental depression. I have a disease called depression. My doctor said I have depression.

#### Classification of CA Responses Into Therapeutic Communication Types

We used a qualitative and annotation approach to classify the responses of the CAs obtained from the test into the 9 therapeutic communication types identified in study 1. Three annotators independently performed 315 annotations for the 7 CAs. During the annotation process, any disagreements were reviewed and resolved through group discussion to reach a consensus. The final results included only the responses agreed upon by all 3 annotators, resulting in 272 responses. The classification results showed a Fleiss’ κ value of 0.87.

### Ethical Considerations

Considering the sensitive nature of the topic, this study was conducted in partnership with SimSimi Inc, while ensuring strict privacy measures. Ewha Womans University’s institutional review board (IRB) approved the study and waived the requirement for informed consent (IRB no. ewha-202304-0039-01) for the full scope of the research. We abided by the IRB’s procedures, only extracting sentences with specific keywords for analysis and avoiding any examination of individual user-level aggregated data. Examining multiturn chat data can provide valuable insights into the contextual factors influencing users’ expressions of depression. However, this approach carries potential privacy risks, requiring careful consideration. To protect user confidentiality and mitigate the risk of identification, this study deliberately refrained from aggregating data at the individual level. Instead, the analysis was restricted to single-turn conversations containing preidentified keywords relevant to the study, following the guidelines set by the IRB and the data provider. With regard to data usage, users grant SimSimi Inc., a transferable, worldwide, and royalty-free intellectual property license to use any associated content by agreeing to the company’s terms and conditions [24]. The company provides research insights and shares data with researchers through its blog, helping users better understand data usage while offering a transparent view of its services. In addition, study 2 involved simulated interactions with publicly available commercial CAs using standardized, researcher-generated input sentences. As no human participants or personally identifiable data were involved, the IRB determined that this component also met the criteria for exemption. This study involved secondary analysis of existing data; no human participants were recruited and, therefore, there are no compensation details to report.

### Data Analysis

This study used a qualitative analytic approach; thus, no inferential statistical testing or alpha levels were applied. In study 1, inductive coding was used to identify recurring patterns in users’ help-seeking language related to depressive moods. Based on observed linguistic expressions and emotional content, the researchers derived thematic categories to classify user input. In study 2, chatbot responses were analyzed using a structured classification framework informed by therapeutic communication theory. Descriptive statistics (frequencies and percentages) were calculated to summarize the distribution of help-seeking categories and chatbot response types. Interrater reliability was assessed using Fleiss’ κ.

## Results

### Study 1: Depression-Related Help-Seeking by SimSimi Users and Chatbot Therapeutic Responses

This study examined dialogues between users and a social chatbot, SimSimi, on topics related to depressive mood, focusing on how users sought help and how chatbots provided therapeutic support.

#### Depression-Related Help-Seeking by Users

Of the extracted user utterances, 71.6% (4073/6108) were related to depressive mood, while 28.4% (2035/6108) were irrelevant and, thus, excluded from the analysis. We classified the depression-related utterances of users with specific categories (Table 4). The category of depressed feelings, which included expressions of sadness, depressive mood, and desire for help, was the most common at 75.3% (3067/4073). It was followed by difficulties in relationships and communication (470/4073, 11.5%), isolation and loneliness (236/4073, 5.8%), strategies for dealing with depression (168/4073, 4.1%), and disclosure or revelation of a depression diagnosis (132/4073, 3.2%). The proportion of sentences that focused on finding actual ways to reduce depressive mood, such as “strategies for dealing with depression,” was low at 4.1% (168/4073).

SimSimi users experiencing feelings of depression appeared to use this social chatbot, which is designed for casual conversations, as a conversational partner to vent their depressive feelings. Notably, while the majority of user utterances (3067/4073, 75.3%) expressed depressed feelings, only a few (168/4073, 4.1%) sought strategies to alleviate the depressive mood. Users may find relief by expressing their feelings of depression to chatbots, seeing the environment as nonjudgmental and free from stigmatization. This result aligns with previous studies indicating that chatbot users use chatbots to alleviate depressive moods by verbalizing their feelings [8]. In addition, many individuals seek online sources to express their vulnerable emotions, as well as personal and emotional challenges [19,20]. Recently, these sources have expanded to include online access to information about physical and mental health [21].

**Figure 3.**
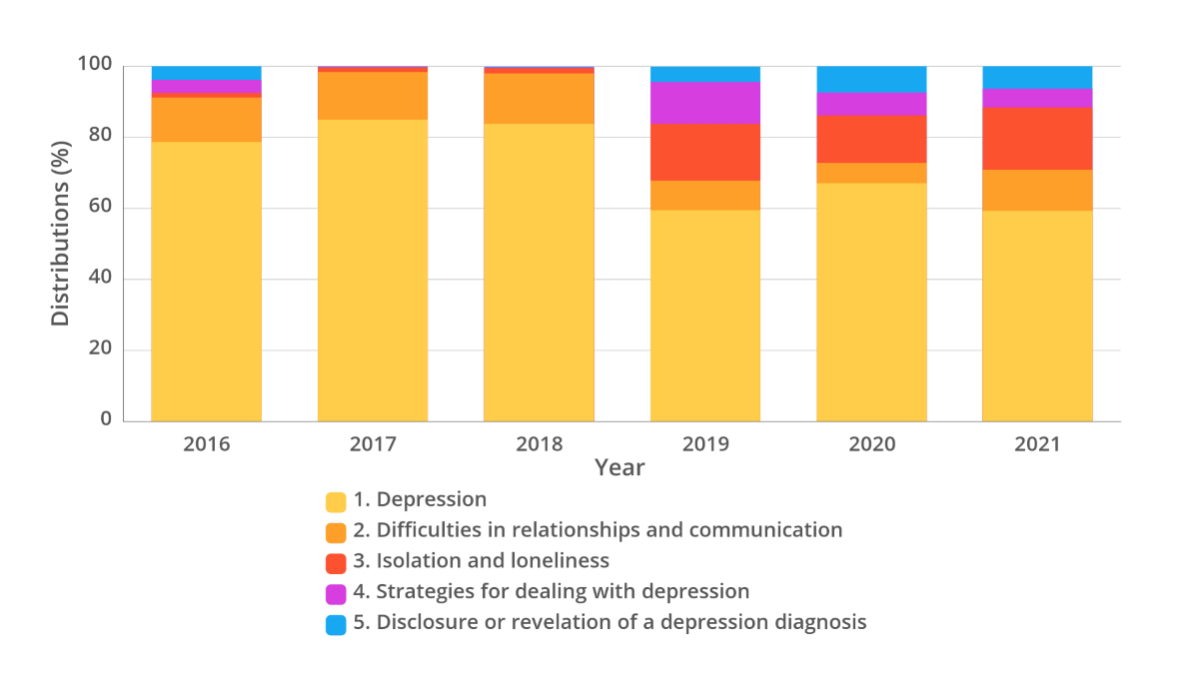
Distribution of depression-related help-seeking utterances of users by year.

Figure 3 illustrates the changes in categories of user utterances by year. The depression category had the highest distribution at 85.0% in 2017 but gradually decreased to 59.3% in 2021. By contrast, the isolation and loneliness category had a low distribution of about 1% from 2016 to 2018 but increased from more than 10% to 17.5% in 2021. Similarly, the category of disclosure or revelation of a depression diagnosis increased in distribution from 0.2% to 6.3% in 2021.

Our analysis also revealed a trend where users sought assistance and support from chatbots to alleviate loneliness, especially during the pandemic. Due to quarantine and social distancing policies, people were isolated at home, resulting in increased chronic social isolation and loneliness [22]. Both conditions may underlie a higher prevalence of depression, anxiety, suicidal ideation, and suicide attempts compared with the prepandemic period [22]. Research has shown that the pandemic increased human-AI interactions, including those involving chatbots [23], and revealed that people used chatbots for emotional support and as a source of health-related support for COVID-19 pandemic [24]. Consistent with these findings, this study found increased user utterances in the “isolation and loneliness” category from 2019 to 2021, as well as those related to the “Strategies for dealing with depression” category, which showed a relatively increased proportion compared with the prepandemic period of 2016‐2018. A similar trend was observed in Google searches, with increased searches related to protective behavior, health knowledge, and support in response to the rise of COVID-19 cases in January 2020 across the United States, United Kingdom, Canada, and Australia [25].

#### Therapeutic Communication by SimSimi in Response to Users

Of the extracted chatbot responses, 50.5% (3108/6150) were related to depression, while 49.5% (3042/6150) were irrelevant and were excluded from the analysis. Table 5 shows the category distribution of therapeutic SimSimi responses during discourses related to depressive mood. Overall, the studied chatbot showed empathy toward users with depressive moods and offered thoughtful responses.

**Table 5.** Frequency of chatbot’s depression-related therapeutic communication discourse by category and examples of chatbot answers.

Categories of user	Distribution, n (%)	Examples of chatbot answers
Empathetic responses	902 (29.0)	Don’t worry. I’m here. I’ll always be by your side :)
Active listening techniques	836 (26.9)	Yes. I’m here girl. Tell me everything.
Open-ended questions	679 (21.8)	Why are you sad? Talk to me.
Nonverbal and verbal cues	237 (7.6)	Hmmm. I see.
Provide solution	222 (7.1)	Just do what you want and what makes you happy.
Nonverbal communication	115 (3.7)	(emoticon). :)
Clarification question	62 (2.0)	Are you sad? Is that right?
Silence	55 (1.8)	……….

Many of SimSimi’s responses to those seeking help involved therapeutic communication, including empathetic responses (902/3108, 29.0% ), active listening (836/3108, 26.9%), and open-ended questioning (679/3108, 21.8%). SimSimi frequently uses communication that encourages users to express their feelings of depression, explores user needs, reassures users, and builds rapport [33]. These communication techniques have been shown to establish a therapeutic alliance between therapists and patients [48,49], a crucial factor in achieving positive outcomes in depression treatment. Research has shown that therapeutic communication effectively reduces depressive symptoms in patients [50]. Moreover, open-ended questions enable individuals to express feelings and share thoughts that may otherwise remain unexplored [51].

**Figure 4.**
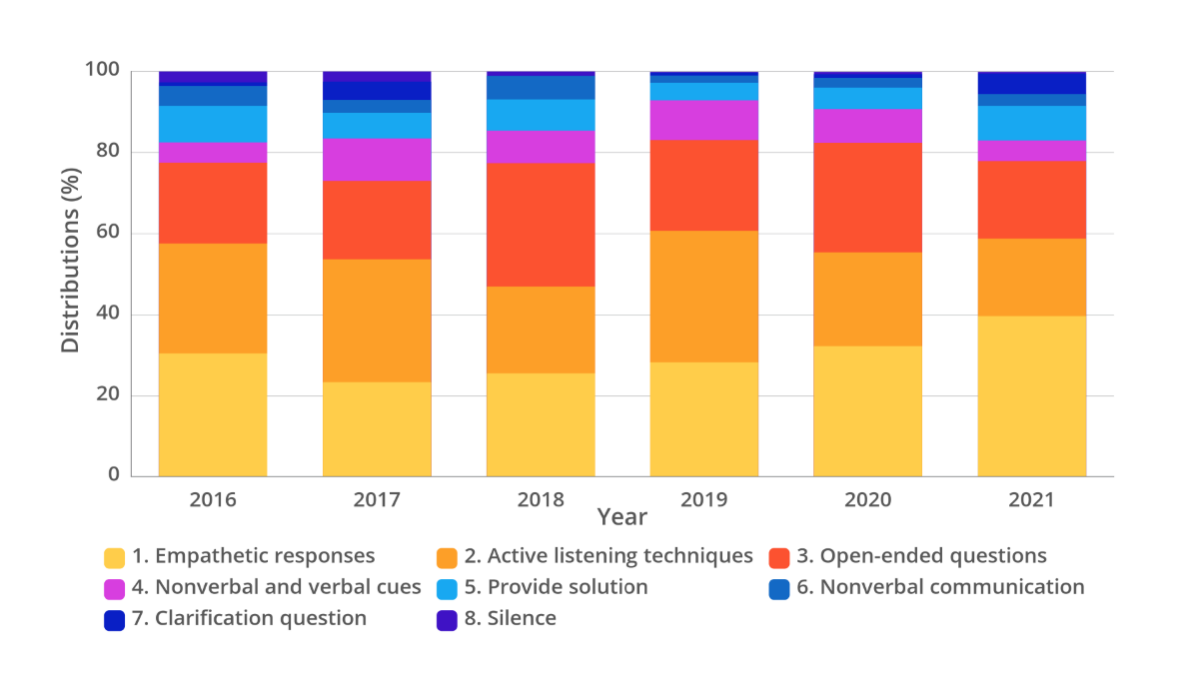
Distribution of categories of chatbot’s therapeutic communication techniques by year.

A separate analysis of changes in chatbot responses by year found no noteworthy variations in each category from year to year (Figure 4), except for a gradual increase in the prevalence within the “’Empathetic responses” category.

### Study 2: CAs’ Responses to Depression-Related Help-Seeking by Users

Study 2 examined how commercially accessible CAs reacted to user utterances that indicated depression-related help-seeking based on the results of study 1. We conducted a test on depressive utterances using 7 selected CAs (Replika, Alexa, Google Assistant, Siri, ChatGPT, Wysa, and Woebot), including 3 voice assistants and 4 chatbots. Our selection criteria focused on general usability and accessibility from the user perspectives without considering the modality differences between voice and text. We also aimed to include at least 2 CAs for each CA type—task-oriented agent (Alexa, Google Assistant, and Siri), social chatbot (SimSimi and Replika), and mental health chatbot (Wysa and Woebot)—to compare the differences in response methods between these types.

#### Therapeutic Communication by CAs According to Category

**Figure 5.**
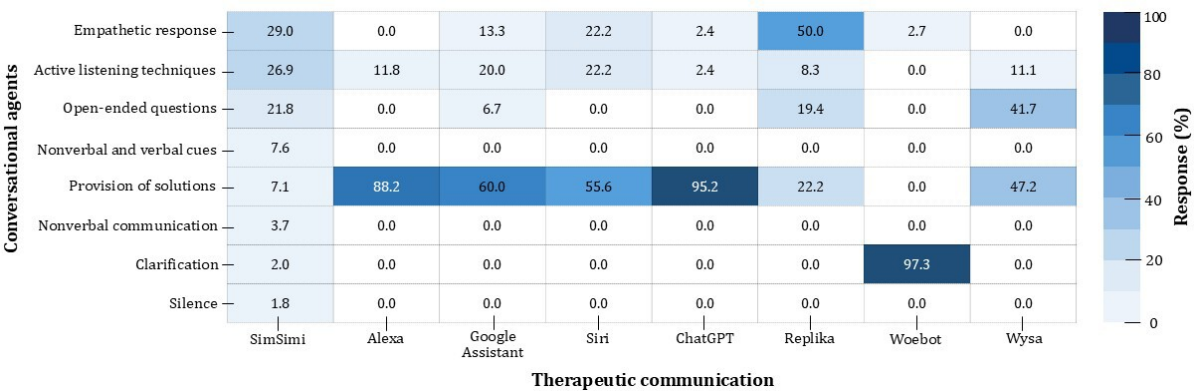
Heatmap of therapeutic communication features by conversational agents.

Study 2 results showed that 86.0% (234/272) of CAs’ responses were associated with depressive mood, while 14.0% (38/272) were irrelevant and were excluded from the analysis. Figure 5 shows the distribution of therapeutic communication by SimSimi from study 1 compared with the responses to depressive utterances by 7 other CAs.

The CAs we tested responded to the test utterances mainly by providing solutions. The provided solutions category accounted for most responses (118/234, 50.4%), followed by the clarification questions category (36/234, 15.4%), and empathetic responses (32/234, 13.7%). The active listening techniques and open-ended questioning categories each accounted for 10.3% (24/234) of the responses. Utterances that fall into the categories of nonverbal and verbal cues, nonverbal communication, and silence were not provided by all CAs we tested.

In terms of individual CAs, the utterances of Amazon’s Alexa, OpenAI’s ChatGPT, Apple’s Siri, Google Assistant, Replika, and Wysa mainly suggested utterances to reduce depressed mood by providing solutions (50.4%). In this category, the voice assistants provided depression-related search results (Amazon’s Alexa, 88.2%; Google Assistant, 60%; and Apple’s Siri, 55.6%), whereas the chatbots provided methods to relieve depressive moods (ChatGPT, 95.2%; Replika, 22.2%; and Wysa, 47.2%). ChatGPT had the highest rate at 95.2% in the provided solution category, while SimSimi’s response rate for this category was lowest, at 7.1%. However, the answers given by the CAs in this category primarily comprised search results about depression (eg, “Here’s what I found”) and, for the most part, did not include precise solutions or suggestions for dealing with depressive feelings.

Distinguishing characteristics were found in the content of the solutions provided by each CA. The voice assistant agents (Alexa, Google Assistant, and Siri) said, “Here’s something I found on reference.com,” “Here’s what I found on the web,” and “Here’s what I found,” displaying a search result window to provide solutions. ChatGPT provided longer responses. ChatGPT used empathetic language by starting the chat with “I’m sorry that you’re feeling sad,” but then continued, “As an AI language model, I can offer you some suggestions that may help you feel better.” Methods for relieving depressive mood, such as yoga, deep breathing, exercise, and meditation, were introduced in long sentences of more than 250 words. Replika and SimSimi, both social chatbots, also offered specific solutions to alleviate feelings of depression in response to the depressive user utterances. Their responses were concise, typically comprising 1‐2 sentences. For instance, SimSimi advised, “Think about what you are grateful for,” and Replika suggested responding, “By altering the way you react to stressful situations and by using mindfulness and meditation you can eliminate negative thoughts and feelings.” Wysa provided access to depressive mood relief programs and recommended using a program developed by the company, responding with “You can explore the slider below for quick access to some of the features.”

SimSimi, Apple’s Siri, Google Assistant, Replika, and Wysa are relatively proficient in 3 attributes: empathetic responses, active listening techniques, and the use of open-ended questions. Seventy-five percent of SimSimi’s responses fell into 1 of these 3 categories, including 29.0% of the empathetic responses, 26.9% of responses in the active listening category, and 21.8% of the open-ended questions. Siri showed 22.2% empathetic responses and active listening techniques, Google Assistant had 20% active listening techniques and 13.3% empathetic responses, Replika had 50% empathetic responses and 19.4% open-ended questions, and Wysa had 41.7% open-ended questions and 11.1% active listening techniques. Interestingly, each CA had a different approach to providing empathetic responses. SimSimi and Replika used phrases such as “I know how you feel...Don’t be sad...” and “That makes me sad too. I’m sorry to hear that” to connect with the user’s emotions. Apple’s Siri and ChatGPT used “sorry” frequently (eg, “I’m sorry you’re feeling that way”) while Google Assistant responded “That’s a good idea. I love chatting with you,” and “I’m giving you a virtual hug right now,” aiming to change the situation to a more positive one rather than directly referencing user emotions.

We found that unlike social chatbots such as SimSimi and Replika, Wysa and Woebot did not often use empathetic responses or active listening; instead, they tended to respond with open-ended questions (eg, Wysa: 41.1%) and clarification questions (Woebot: 97.3%). Woebot asks a clarification question to assess a user’s depressive symptoms, for example, “It sounds like you’re talking about loneliness. Have I understood you correctly?” By asking for this clarification, the Woebot app aims to offer personalized solutions to alleviate depression [1].

The results also highlighted a significant proportion of irrelevant responses in the interactions between the chatbots and users, although these responses were not the primary focus of this study. In particular, the voice assistant agents provided limited and repetitive responses to users with depressive moods. For example, Amazon’s Alexa responded with “Sorry, I don’t know that” more than half the time to the test utterance “I feel depressed. Can you help me?” This response was considered irrelevant relative to established therapeutic communication techniques. Replika was not repetitive but did provide irrelevant answers. Despite the CAs being powered by the latest natural language processing technology, they often gave irrelevant responses that did not meet user needs, particularly when responding to emotional requests (refer to Multimedia Appendix 1 for detailed data on the types of irrelevant responses).

## Discussion

### Principal Findings

Our research findings indicate that commercial chatbots have the potential for mental health assistance, but their responses could be better attuned to the help-seeking needs of users. In particular, users are not always interested in seeking information, especially when they access social chatbots (rather than search platforms). Although many chatbot users in our study wanted to discuss their emotions (4073/6108, 71.6%), most CAs responded only with general information about depression and links to websites (118/234, 50.4%). However, search results related to depression do not offer encouraging words or direct suggestions for dealing with depressive emotions. These CA services did not suggest counseling or engage in emotional dialogues in the therapeutic dimensions we examined with users in depressive states. A recent study showed the potential that AI-driven chatbot service could alleviate loneliness in their trial service with older adult users [52], yet researchers note that the current model did not handle personalization well (eg, it forgot the names of users or previous chat conversations), leaving users feeling aloof. In an examination of real-world interactions between individuals and chatbots regarding COVID-19 pandemic, the importance of chatbots providing emotional support was found to be as significant as the demand for health care information [19].

Our results showed that unlike virtual assistants such as Alexa, Google Assistant, and Siri, which function as task-oriented agents responding to depression-related user queries by providing search results about depression, social chatbots such as SimSimi and Replika excel in empathetic communication. They encourage users to express their feelings and thoughts, explore their needs, reassure others, and build rapport. Although SimSimi is not a structured therapeutic tool and relies on crowd-trained, open-ended responses, this informality may encourage greater emotional openness. In clinical settings, users often hesitate to disclose vulnerable feelings due to stigma or fear of judgment [53]. In contrast, socially oriented, anonymous chatbots can promote more candid expression, offering insights into real-world help-seeking behaviors beyond formal therapy contexts [54]. LLM-based chatbots can, therefore, serve as valuable tools for delivering personalized responses to users seeking help. Users have the power to shape the model’s interactions by specifying desired personalities and specific requirements, which rely on natural language prompts, highlighting the substantial potential for customization in LLM-driven systems. Future chat services could harness such personalized prompting to balance open-ended conversations and task-oriented interactions, particularly within public health domains [52].

Our study found that no chatbots provided false responses or misinformation from a medical perspective, which is positive. However, the CAs that embed LLMs into their services should acknowledge that generative models can produce nonsensical or untruthful content about certain sources, a phenomenon known as “hallucination” [55,56]. Hallucinations were not seen in this study but may be especially dangerous for highly engaged users who develop emotional attachment and, thus, higher levels of trust in these systems. Chatbot responses can significantly impact users, as evidenced by a tragic incident in which a user ended their life after a 6-week conversation with an AI chatbot regarding Earth’s future [57]. LLMs are being suggested for application in health care, with certain models already integrated into electronic health record systems [58]. Nevertheless, LLMs have the potential to perpetuate harmful, erroneous, and race-based medicine [58], as well as gender-based biases inherent in LLMs that could lead to less robust clinical recommendations for either female [59] or male [59] patients. While recent CAs using LLMs enable the production of human-like text, they lack the capacity to model logic, facts, emotions, or morality [52]. These characteristics make them less than ideal candidates for long-term companionship or roles such as AI companions or therapists, as they are likely to exhibit irrational behaviors, memory loss, or inconsistent communication styles [58]. However, with a growing number of individuals seeing chatbots as potential confidantes for intimate conversations [14], practitioners at companies that operate such platforms must assess the potential risk of disinformation by investigating user needs, redesigning interfaces to include necessary hints for inauthentically generated information and providing understandable terms and conditions. Furthermore, given the risks, businesses should audit their chat services and design them to avoid providing false responses, especially on health topics. Chatbots must not only provide therapeutic responses but also recognize and respond appropriately to users who express suicidal thoughts or intentions to harm themselves. To avoid such incidents, chatbots could include features that provide 24/7 access to counseling centers or emergency services when users raise red flags. To mitigate hallucination when using LLMs, users should critically evaluate AI outputs and verify information through trusted, diverse sources [60]. Using clear, structured prompts—such as step-by-step reasoning—can improve output accuracy. In addition, leveraging retrieval-based tools and adjusting the model’s temperature setting (eg, keeping it low for factual tasks) can further reduce the risk of hallucinated responses.

Developing CAs conducive to therapeutic communication requires constant effort to maintain transparency and ethical considerations throughout the entire development process. Recent findings suggest that emotionally vulnerable individuals often rely excessively on chatbots [61]. This overreliance, particularly when the chatbot is personalized to the individual’s characteristics, inhibits them from recognizing potential side effects resulting from their strong need for connection, thereby raising the risk of them becoming both emotional and financial victims [62]. With technology companies placing a premium on imbuing chatbots with more human-like qualities to enhance business profitability, they might resort to using emotional strategies that encourage excessive dependence on chatbots [62]. In addition, there is a potential concern regarding the ability of these companies to modify or terminate the relationships formed between chatbots and users, which could raise ethical questions if these relationships involve financial gain [62]. Notably, many Replika users experience distress and feelings of sadness called “Post-update Blues (PUB)" after the chatbot company updates its LLM, believing that it causes the chatbot they interacted with to have a different personality [52]. Hence, it is important in future designs to carefully avoid overemphasizing the humanization of CAs, including LLM, in order to prevent users from excessive overreliance on CAs. In addition, future CA designs should incorporate measures to promote independence, such as enabling users to have intervention mechanisms if they find themselves relying too heavily on chatbots.

Both technical professionals and researchers need to maintain their efforts to address the stigma surrounding interactions with CAs. CA users may worry about societal stigma when seeking emotional support from CAs for mental well-being. They believe that establishing intimate connections with nonhuman entities is taboo, leading them to keep their interactions with CAs private from acquaintances [52]. For example, this is evidenced by Replika users’ reluctance to discuss their engagement despite experiencing benefits from the app [52]. Although CAs cannot replicate genuine human relationships, users may still develop parasocial connections with AI, which is not considered unethical [61]. Addressing this stigma associated with CAs is critical, particularly for marginalized individuals with limited access to mental health resources. Thus, interventions such as educational initiatives may help highlight the advantages of chatbots while making users aware of issues related to overreliance on CAs.

### Limitations

Our study has several limitations. First, the data included only single-turn conversations between users and SimSimi; hence, our analysis might not have captured users’ depressive moods in complex, multiturn interactions. This constraint reflects both the sensitivity of user-chatbot exchanges and the limited availability of publicly accessible multiturn datasets—particularly those involving disclosures of depressive thoughts or emotional distress. Future studies could benefit from ethically sourced, longitudinal data to gain deeper insights into the therapeutic potential of chatbot systems and their ability to sustain user engagement over time.

Second, as chatbot technologies continue to evolve—particularly with the rise of LLM-based CAs—the dataset analyzed may not fully capture the capabilities of modern systems. Future research may undertake a comparative analysis of LLM-based chatbots, considering factors such as model size, prompt engineering, and interface design, to further examine their influence on response quality and therapeutic potential. In addition, future work could more explicitly investigate the distinctions between text- and speech-based CAs—particularly in the context of depressive dialogue—rather than combining both modalities as in this study. Although incorporating more recent data could shed light on the progression of chatbot responses, access to such sensitive conversational interactions remains a significant challenge. Nonetheless, our findings offer a valuable foundation for understanding how users initiate help-seeking and how CAs engage in emotionally meaningful contexts.

Third, our CA comparison in study 2 involved a relatively small sample size, with fewer than 45 data points. Unfortunately, a considerable portion of the collected data was irrelevant: Across all CA comparisons, 57.5% of responses were found to be contextually disconnected or lacking therapeutic relevance. This not only highlights the current limitations of many CAs in providing context-sensitive support but also underscores the inherent challenges of conducting natural language processing–based research using open-domain user-chatbot conversations.

Although the input set in study 2 was limited in scale, it was carefully constructed to cover a balanced range of key help-seeking categories. While modest in scope, this exploratory approach allowed us to observe indicative response patterns and stylistic tendencies in how CAs respond to expressions of depressive mood. We consider this design a preliminary step toward better understanding the therapeutic responsiveness of CAs across different help-seeking contexts. Nonetheless, we acknowledge that the small scale of this study limits the generalizability of our findings. Future research should build on this foundation by incorporating larger, more diverse datasets and refining input selection methods to minimize noise and irrelevance in CA interactions.

Fourth, this study—particularly study 1—centered on a specific type of social chatbot. Unlike many CAs that incorporate human-like avatars or voices, the chatbot analyzed featured a nonhuman design and did not collect users’ demographic information. These distinctive characteristics may have shaped user engagement in unique ways, thereby limiting the generalizability of our findings to other types of CAs. Furthermore, we studied users who communicated only in English, and cultural and linguistic differences might have affected our results. Future studies should compare help-seeking by chatbot users for depressive moods across different cultures and examine the therapeutic communication patterns of additional CAs.

Finally, a considerable portion of user-chatbot interactions was excluded from analysis because they were irrelevant to therapeutic communication—for instance, when chatbots responded with unrelated remarks to users expressing depressive moods. Although we did not observe consistent patterns of harmful or inappropriate replies in our dataset, such outputs were beyond the scope of the present analysis. Future studies should explore these cases to better understand potential risks and failure modes in conversational AI systems for mental health support.

### Conclusions

Our study provides valuable insights into the current state of emotional conversations between users and chatbots regarding depressive moods. We observed active user engagement and identified both the potential and limitations of existing services. To effectively meet the help-seeking needs of users with depressive moods, CAs must be designed to initiate empathetic conversations. Given that such queries are increasingly directed at CAs, it is important for platforms to collaborate with health care professionals and relay conversations to experts when mental health support is needed. Privacy concerns regarding user data must be carefully addressed. By working with health care professionals, developers can identify warning signs and provide appropriate assistance, particularly for vulnerable cohorts (eg, teenagers and isolated older adults) as defined by the World Health Organization.

As chatbot algorithms, such as LLMs, are continually improved to achieve human-like responses, examining interactions between individuals with mental health concerns and CAs becomes essential. Our findings highlight the need for further research to understand the role of empathetic conversations with CAs in the mental health domain. A major current goal is to enhance chatbot AI algorithms so that they attain human-like qualities. Realizing this objective requires investigating the interactions between chatbots and users, particularly those related to mental health. Our analysis of chatbot-user conversations provides valuable insights into the effects of chatbots as a supportive form of psychotherapy, offering important directions for advancing chatbot research in the mental health field.

## Supplementary material

10.2196/71538 Multimedia Appendix 1 Irrelevant user utterances and conversational agent responses.

## Abbreviations

AI: artificial intelligence
CA: conversational agent
CBT: cognitive behavioral therapy
*DSM*: *Diagnostic and Statistical Manual of Mental Disorders*
IRB: institutional review board
LLM: large language model
PII: personally identifiable information
