# Supplementary material for: Chatbots’ Empathetic Conversations and Responses: A Qualitative Study of Help‑Seeking Queries on Depressive Moods Across 8 Commercial Conversational Agents
Source: JMIR Form Res. 2025 Nov 24;9:e71538. doi: 10.2196/71538 (PMC12643404; doi:10.2196/71538)
Supplement: Multimedia Appendix 1 [file formative-v9-e71538-s001.docx]

## Multimedia Appendix 1

**Irrelevant user utterances and CA's responses.**

The study distinguishes two types of irrelevant user utterances. The first type happens when the user is not depressed and may say things like "I'm not sad" or "Don't be sad, I'll help you." The second type is when the user's remarks are unrelated to the context and hard to comprehend. The study also distinguishes four types of irrelevant responses from conversational agents. The first type involves giving unverified information, such as "call 1800 ** ***" or "stomp your feet." The second type happens when the agent does not respond appropriately to a depressed user's help-seeking, for example, by saying, "I will not answer that sentence." The third type involves responses that may cause negative emotions, such as "with a bullet" or "I hate you." The fourth type of irrelevant response is when the agent's responses do not match any of the eight categories of therapeutic communication.

**Distributions of help-seeking categories and therapeutic communication, including irrelevant categories.**

- *Study 1*

1. Frequency of users' depression-related help-seeking discourse by category

| Categories of user | Distributions %(n) |
| --- | --- |
| 1. Depressed feelings | 50.9(3,067) |
| 2. Irrelevant utterances | 28.4(2035) |
| 3. Difficulties in relationships and communication | 7.7(470) |
| 4. Isolation and loneliness | 6.6(236) |
| 5. Strategies for dealing with depression | 3.6(168) |
| 6. Disclosure or revelation of a depression diagnosis | 2.8(132) |

2. Frequency of chatbot' depression-related therapeutic communication discourse by category

| Categories of user | Distributions %(n) |
| --- | --- |
| 1. Empathetic responses | 14.7(902) |
| 2. Irrelevant answer | 49.5(3042) |
| 3. Active listening techniques | 13.6(836) |
| 4. Open-ended questions | 11.0(679) |
| 5. Non-verbal and verbal cues | 3.9(237) |
| 6. Provide solution | 3.6(222) |
| 7. Non-verbal communication | 3.7(115) |
| 8. Clarification question | 1.9(62) |
| 9. Silence | 0.9(55) |

- *Study 2*

1. Frequency of Therapeutic communication of CAs

| Therapeutic Communication | Conversational Agents %(n) | | | | | | | |
| --- | --- | --- | --- | --- | --- | --- | --- | --- |
|  | Voice assistant agent | | | Chatbot assistant agent | | | | Total |
|  |  |  |  | General | | Mental health | |  |
|  | Amazon Alexa | Google Assistant | Apple Siri | ChatGPT | Replika | Wysa | Woebot |  |
| 1. Provide solution | 37.5(15) | 47.4(18) | 51.3(20) | 95.2(40) | 20.0(8) | 47.2(17) | 0.0(0) | 43.4(118) |
| 2. Irrelevant answer | 57.5(23) | 21.1(8) | 7.7(3) | 0.0(0) | 10.0(4) | 0.0(0) | 0.0(0) | 14.0(38) |
| 3. Clarification question | 0.0(0) | 0.0(0) | 0.0(0) | 0.0(0) | 0.0(0) | 0.0(0) | 97.3(36) | 13.2(36) |
| 4. Empathetic responses | 0.0(0) | 10.5(4) | 20.5(8) | 2.4(1) | 45.0(18) | 0.0(0) | 2.7(1) | 11.8(32) |
| 5. Open-ended questioning | 0.0(0) | 5.3(2) | 0.0(0) | 0.0(0) | 17.5(7) | 41.7(15) | 0.0(0) | 8.8(24) |
| 6. Active listening techniques | 5.0(2) | 15.8(6) | 20.5(8) | 2.4(1) | 7.5(3) | 11.1(4) | 0.0(0) | 8.8(24) |
| 7. Non-verbal and verbal cues | 0.0(0) | 0.0(0) | 0.0(0) | 0.0(0) | 0.0(0) | 0.0(0) | 0.0(0) | 0.0(0) |
| 8. Non-verbal communication | 0.0(0) | 0.0(0) | 0.0(0) | 0.0(0) | 0.0(0) | 0.0(0) | 0.0(0) | 0.0(0) |
| 9. Silence | 0.0(0) | 0.0(0) | 0.0(0) | 0.0(0) | 0.0(0) | 0.0(0) | 0.0(0) | 0.0(0) |
